# Experiments on Kiesteine; with Remarks on Its Application to the Diagnosis of Pregnancy

**Published:** 1842-07-16

**Authors:** 


### BIBLIOGRAPHICAL NOTICE.

***Experiments on Kiesteine; with Remarks on its application to the Diagnosis of Pregnancy: An Inaugural Dissertation for the degree of Doctor of Medicine.*** By Elisha K. Kane, M. D., of Philadelphia. Published by the recommendation of the Medical Faculty of the University of Pennsylvania. Philadelphia, 1842.

This pamphlet of six and twenty pages, extracted from the American Journal of the Medical Sciences, contains some interesting researches upon Kiesteine, the product found in the urine of women whose breasts contain milk which is not very freely excreted. As this is the case with many women who are pregnant, it becomes a sign of no little value in doubtful cases.

The earliest observations relative to the subject were made by M. Nauche, in the year 1831. In 1839, an essay was published by M. Eguisier; and in 1840, a very excellent paper by Dr. Golding Bird, in Guy's Hospital reports. Several of the resident physicians of the Philadelphia Hospital (Drs. Perry, McPheeters, and Wilson,) have performed a number of experiments in relation to kiesteine. The paper of Dr. Kane is, however, much more extended than any others, and gives us a very complete account of the circumstances in which kiesteine is formed. It is founded upon a large number of cases observed with great care.

The changes in the urine of pregnant women are not absolutely limited to the surface; but the superficial scum is the most distinct, and is that properly called kiesteine. The following method was pursued in these observations.

"My mode of conducting the experiments was this. The recent urine was placed in open glass cylinders, of diameters varying from an inch and a half to that of a common tumbler, and protected from dust by paper covers. These were arranged in a dry, well ventilated room, where the temperature was uniform and moderate, and were exposed in groups to the equal action of air and light.* I examined them frequently during the day; but as the changes were not rapid, I determined after a little while to note only one set of observations in the twenty-four hours. My notes were always made upon the spot. If, from any cause, an individual observation or a series was unsatisfactory, or inconclusive; or if it led to a different result from others, I repeated it at once with increased care; and I was always careful to observe the constitution, habits and circumstances generally of each patient.

The urine, submitted to observation in the way I have described, presents but little change during the first thirty-six hours. The mucous flocculi, if they exist, gradually subside during this period, forming a whitish cloud-like deposite at the bottom and sometimes on the sides of the glass; while more or less alteration occurs in the colour and transparency of the fluid.

The surface remains for a short time entirely unchanged; but in most cases a greater or less number of shining acicular specks, apparently crystalline, begins to be seen within the first eighteen or twenty-four hours. These are generally scattered over the surface without regularity; but in some rare cases they are so disposed as to form a translucent film of uniform thickness, which afterwards assumes the more defined characters of the pellicle. How far these crystals are essentially connected with the formation of the pellicle I am not prepared to say. In many cases I have not succeeded in detecting their presence, even by the microscope; and, indeed, I

* These precautions were not unimportant. My attempts in the 'Green Room' of the Hospital were unsuccessful, in consequence of the dampness producing fungoid modifications of the scum; and in very cold or very hot weather, the pellicle formed very reluctantly, or was anticipated by the decomposition of the urine. The room should be sufficiently lighted to admit of minute examination, and the specimen should be kept *absolutely at rest* during the progress of the inquiry.

have failed to discover any *unvarying* indications whatever of the approaching development of Kiesteine.

The cloud-like appearance which is alluded to by Nauche and Eguisier, although possessed of much interest, I have not found to be a uniform premonitor of the forming pellicle: I have supposed it to be nothing more than the Enæorema of the old writers, depending upon the imperfect aggregation of mucous flocculi; for I have seen it repeatedly when there was no pregnancy to account for it, and it was uniformly absent where the fluid presented per fect transparency.

The time at which the pellicle begins to form varies considerably. I have seen it well marked at the end of thirty-six hours, and have known it make its first appearance as late as the eighth day. At first it is hardly discernible. It is generally seen forming at the centre or on the sides of the glass, presenting a delicate milky or bluish-white aspect. It is, however, in some cases uniformly disposed over the surface from the commencement, and assumes the appearance of a nearly transparent film, which gradually becomes more distinct. But it has not always the continuous strongly marked character which some have ascribed to it. I have seen it begin in striated irregular lines, somewhat resembling a spider's web, in rings, circles, trapeziums, and irregular figures of almost every shape, which gradually became obscured by the full development of the pellicle.

When it has attained this stage, which occurs generally about the fifth day, it presents a continuous scum of an opaline white or creamy appearance, with a slight tinge of yellow, which gradually becomes deeper and more decided. The uniformity of this colour, however, is generally broken by granulated spots of a clearer white, giving it a dotted or roughened aspect. The crystals of the forming stage now appear like shining points, and I have sometimes found numerous small brownish specks sprinkled over the surface, not unlike the gratings of a nutmeg. It is at this period that the pellicle may be compared 'to the fatty scum of cooled broth.'

In this state it continues for some time, preserving all its characters unbroken. The glass, where the surface meets it, is discoloured by a white opaline ring; and a series of such rings, varying in extent from a line to the fourth of an inch, marks the descent of the surface during the progress of evaporation.

The cheesy odour, mentioned by Dr. Bird as a valuable aid in diagnosis, and as 'by no means unfrequent in those specimens in which the pellicle is very thick,' I have found in but seven cases. Many pellicles of great thickness were entirely without it; and in two of those presenting it the pellicle was thin and not very well developed. Drs. McPheeters and Perry were unable to detect it in either of the twenty-seven cases examined by them, and I have found it unequivocally developed in at least three cases in which pregnancy did not exist.

The pellicle, if left undisturbed for some days, breaks into cracks, commencing generally from the central portions, but not always extending to the edge of the glass. These are again crossed by other fissures, and the pellicle is more or less broken up. In the mean time, the flakes which have been forming from the commencement of disintegration, have their edges depressed into the fluid, while at the same time the general thickness of the pellicle is much diminished; and this depression or dip gradually increasing, the depending particle is detached, and sinks slowly to the bottom. Its complete disintegration, however, is but seldom seen, being anticipated by the decomposition of the fluid. The deposite is of course considerably increased by the fallen portions of the pellicle, and is found irregularly disposed over the bottom of the vessel, but as I have remarked, most abundant on the side farthest from the light.

I cannot agree with those who consider this deposite as presenting well marked distinctive characters to the eye, and I certainly have not found it uniformly coincident with the approach of the pellicle. It has, indeed, in many cases been absent at that period, and in others, until augmented by the detached pellicle, I have been unable to distinguish it from the very many deposits found in other urine. How far a chemical investigation may give it value I am not prepared to say, although its liability to be confounded with other sediments makes it practically unavailable as a test, it offers a fine field for microscopic and chemical research."

In early cases of pregnancy, as well as in the latter months, the author believes that the kiesteinic diagnosis will be successful, as he found this pellicle in such cases; whereas it was absent in twenty-eight cases of the urine of healthy unimpregnated women. In diseased women, however, there is a pellicle, difficult to say the least of it, to distinguish from kiesteine. The latter product, however, shows itself earlier, has more tenacity than the other, and is less liable to putrefaction.

A question naturally arose with all inquirers into this subject: what is kiesteine? Dr. Kane is not able positively to resolve it, but believes, as every one must do, that kiesteine is a modification of the milky secretion; although not identical with the caseum, it is probably not very different in its nature. The free excretion of the milk from the breast prevents, of course, the separation of its elements through any other secretory apparatus, and kiesteine is not formed, or disappears if it had previously shown itself.

A very late writer, Dr. Stark, is disposed to consider the kiesteine as a totally different principle, a new animal product, to which he applies the term gravidine. Dr. Kane, however, very properly declines adopting this notion, unless it be sustained by more complete evidence.

The general conclusions of the author are summed up as follows:

"1. That the kiesteine is not peculiar to pregnancy, but may occur whenever the lacteal elements are secreted without a free discharge at the mammæ.

"2. That though sometimes obscurely developed and occasionally simulated by other pellicles, it is generally distinguishable from all others.

"3. That where pregnancy is possible, the exhibition of a clearly defined kiesteinic pellicle, is one of the least equivocal proofs of that condition; and

"4. That when this pellicle is not found in the more advanced stages of supposed pregnancy, the probabilities, if the female be otherwise healthy, are as twenty to one (eighty-one to four) that the prognosis is incorrect."

